# Characterization of a β-carotene isomerase from the cyanobacterium *Cyanobacteria aponinum*

**DOI:** 10.1098/rstb.2023.0360

**Published:** 2024-09-30

**Authors:** Derry Alvarez, Yu Yang, Yoshimoto Saito, Aparna Balakrishna, Kosuke Goto, Takashi Gojobori, Salim Al-Babili

**Affiliations:** ^1^ The BioActives Lab, Center for Desert Agriculture, King Abdullah University of Science and Technology (KAUST), Thuwal 23955-6900, Saudi Arabia; ^2^ Plant Science Program, Biological and Environmental Science and Engineering (BESE) Division, King Abdullah University of Science and Technology (KAUST), Thuwal 23955-6900, Saudi Arabia; ^3^ Marine Open Innovation (MaOI) Institute, 9-25 Hinodecho, Shimizu-ku, Shizuoka 424-0922, Japan; ^4^ Computational Bioscience Research Center (CBRC), King Abdullah University of Science and Technology (KAUST), Thuwal 23955-6900, Saudi Arabia

**Keywords:** strigolactones, carotenoids, cyanobacteria, DWARF27 β-carotene isomerase

## Abstract

Carotenoids are essential components of the photosynthetic apparatus and precursors of plant hormones, such as strigolactones (SLs). SLs are involved in various aspects of plant development and stress-response processes, including the establishment of root and shoot architecture. SL biosynthesis begins with the reversible isomerization of all-*trans*-carotene into 9-*cis*-β-carotene, catalysed by DWARF27 β-carotene isomerase (D27). Sequence comparisons have revealed the presence of D27-related proteins in photosynthetic eukaryotes and cyanobacteria lacking SLs. To gain insight into the evolution of SL biosynthesis, we characterized the activity of a cyanobacterial D27 protein (*Ca*D27) from *Cyanobacterim aponinum*, using carotenoid-accumulating *Escherichia coli* cells and *in vitro* enzymatic assays. Our results demonstrate that *Ca*D27 is an all-*trans*/*cis* and *cis*/*cis*-β-carotene isomerase, with a *cis*/*cis* conversion preference. *Ca*D27 catalysed 13-*cis*/15-*cis-*, all-*trans*/9-*cis*-β-carotene, and neurosporene isomerization. Compared with plant enzymes, it exhibited a lower 9-*cis*-/all-*trans*-β-carotene conversion ratio. A comprehensive genome survey revealed the presence of *D27* as a single-copy gene in the genomes of 20 out of 200 cyanobacteria species analysed. Phylogenetic and enzymatic analysis of *Ca*D27 indicated that cyanobacterial *D27* genes form a single orthologous group, which is considered an ancestral type of those found in photosynthetic eukaryotes.

This article is part of the theme issue ‘The evolution of plant meta‌bolism’.

## 1. Introduction

Carotenoids are essential isoprenoid photosynthetic pigments with diverse roles in light-harnessing and photoprotection [1,2]. In plants, they play an important role as precursors for hormones and different signalling molecules, such as abscisic acid (ABA), strigolactones (SLs), zaxinone, anchorene and β-cyclocitral [3–7]. These compounds originate through oxidative reactions triggered by reactive oxygen species (ROS) or the carotenoid cleavage dioxygenase protein family (CCDs), leading to the generation of diverse compounds called apocarotenoids (for review, see [3,4,8]). Different enzymes frequently modify primary cleavage products before gaining regulatory activity. ABA biosynthesis results from a series of reactions involving cleavage of carotenoids, transforming 9-*cis*-violaxanthin or 9′-*cis*-neoxanthin into xanthoxin [9,10]. In SL biosynthesis, all-*trans*-β-carotene is isomerized to 9-*cis*-β-carotene, by DWARF27 isomerase (D27) [11]. Subsequently, 9-*cis*-β-carotene is cleaved by CCD7, a stereospecific enzyme that accepts only 9-*cis*-configured carotenoids, at the C9′–C10′ double bond to produce 9-*cis*-β-apo-10′-carotenal and β-ionone [11–15]. CCD8 then converts the 9-*cis*-β-apo-10′-carotenal product into the principal intermediate in SL biosynthesis, carlactone (CL), which is the simplest non-canonical SL [3,14,16]. Additionally, a recently characterized D27 homologue, D27-Like1, catalyses all-*trans*/*cis* and *cis*/*cis* conversions of β-carotene, contributing to SL biosynthesis [17].

SLs were discovered as germination stimulants in root-parasitic plants and were later identified as mediators in establishing arbuscular mycorrhizal (AM) symbiosis [18,19]. SLs are important plant hormones involved in various aspects of plant developmental processes, including the determination of root and shoot architecture, secondary growth, and responses to biotic and abiotic stresses [3,4,8,20,21]. Structurally, SLs are characterized by a conserved methylbutenolide ring (D-ring) in the *R* configuration, attached by an enol bridge to a second moiety with various structures [4]. Depending on the structure of this moiety, SLs can be divided into canonical and non-canonical types [14]. Recent findings indicate that this structural diversity is linked to specific functions [11,16,22].

However, the evolution of SL biosynthesis remains unclear. The presence of diverse genes appearing at different points in the evolutionary record complicates the reconstruction of this process [8,23,24]. Previous studies investigating the phylogenies of D27, CCD7 and CCD8 have provided insights into the emergence of SL biosynthesis, suggesting that the SL core (*D27*, *CCD7* and *CCD8*) are ancestral in land plants [8,23–25]. The lack of experimental data supporting the assumed functions of genes identified based only on sequence homology, and the exclusive dependency on *in silico* analysis, make it difficult to understand the evolutionary history of SLs. The only evidence to date regarding an evolutionarily ancient SL is the identification of bryosymbiol, an SL from the bryophyte *Marchantia paleacea* [26]. Interestingly, to date, no studies have been performed on D27 homologues from SL-lacking organisms, despite their widespread presence, even in some cyanobacteria.

In addition to D27 and D27-Like1, sequence comparisons revealed the presence of another D27 homologue, D27-Like2 [17,25,27,28]. Phylogenetic analysis classified the D27 protein family into three clades. The first two clades (I and II) consisted of only representatives from land plants, whereas the third clade (III) included enzymes from chlorophyte algae and diatoms [25]. Although the sequences of *D27* in cyanobacteria have been reported, the phylogenetic relationship between cyanobacterial *D27* and photosynthetic eukaryotes in Archaeplastida remains unclear.

D27 catalyses the reversible 9-*cis*-/all-*trans*-isomerization of β-carotene, with an equilibrium in favour of the all-*trans*-isomer [12,28]. However, the formation of 9-*cis*-β-carotene is an indispensable step in SL biosynthesis. *Arabidopsis* and rice *d27* mutants showed mild SL-deficiency phenotypes compared with *ccd7* and *ccd8* mutants, suggesting that D27 activity can be exerted by another enzyme or a non-enzymatic process [25]. To date, only the D27 (rice, *Arabidopsis* and saffron) and D27-Like1 (*Arabidopsis*) have been studied in detail [11,12,17,28,29]. Enzymatic studies of *Arabidopsis* D27-Like1 demonstrated its capability to catalyse *cis*/*cis* and all-*trans*/*cis* conversions and the formation of 9-*cis*-β-carotene [17,27], indicating a contribution to SL biosynthesis. This assumption was confirmed by characterizing the *Arabidopsis d27*/*d27-like* double mutant and by restoring the *Arabidopsis d27* phenotype through ectopic expression of *D27-Like1* [17].

In the present study, we investigated the enzymatic activity of *Cyanobacterium aponinum* D27 (*Ca*D27). Our results demonstrated for the first time that cyanobacterial D27 catalyses *cis*/*cis*-isomerization reactions and all-*trans*-/*cis* conversions. Additionally, we conducted genomic surveys of cyanobacteria and investigated the distribution of *D27* genes within this taxon. Furthermore, we conducted a phylogenetic analysis using sequences of cyanobacterial D27 proteins together with those from Rhodophyta, Chlorophyta and Streptophyta, including algae (*Klebsormidium*) and land plant species, revealing orthologous relationships among them. The results suggest that cyanobacteria *D27* genes are ancestral types of β-carotene isomerases of photosynthetic eukaryotes, providing new insights into the evolution of D27 enzymes and the first step in SL biosynthesis.

## 2. Results

### (a) Recombinant *Ca*D27 catalyses all-*trans*/*cis*, and *cis*/*cis* isomerization of carotenes *in vitro* and *in vivo*

Carotenoid-accumulating *E. coli* strains are efficient systems for characterizing carotenoid-metabolizing enzymes. Therefore, we studied the expression of *Ca*D27 protein fused to thioredoxin, encoded by pThio-*Ca*D27, in β-carotene-, lycopene-, zeaxanthin- and neurosporene-accumulating *E. coli* cells [30,31]. The introduction of *Ca*D27 into β-carotene-accumulating *E. coli* cells caused an increase in the 9-*cis*/all-*trans*-β-carotene ratio (electronic supplementary material, figure S1a). Additionally, this enzyme isomerized all-*trans*-neurosporene into 9-*cis*- and 15-*cis*-neurosporene (electronic supplementary material, figure S2a,b). In contrast, we did not observe any enzymatic activity in all-*trans*-lycopene- and all-*trans*-zeaxanthin-accumulating *E. coli* cells (electronic supplementary material, figure S1b,c).

**Figure 1.**
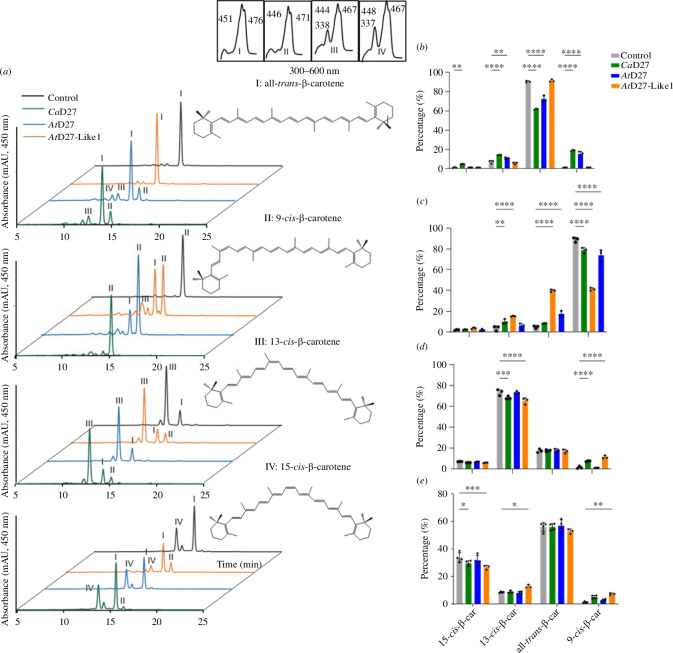
Ultra-high performance liquid chromatography (UHPLC) analysis of *in vitro* assays performed with crude lysates of BL21(DE3) *Escherichia coli* cells expressing thioredoxin-*Ca*D27 (*Ca*D27), thioredoxin-*At*D27 (*Arabidopsis thaliana* D27), thioredoxin-*At*D27Like1 (*At*D27-Like1) or thioredoxin (Control) with different β-carotene isomers. Left: (*a*) chromatograms of the incubations with different β-carotene isomers. Right: the relative peak surface of the different β-carotene isomers separated in the chromatograms; (*b*) all-*trans*-β-carotene (peak I); (*c*) 9-*cis*-β-carotene (peak II); (*d*) 13-*cis*-β-carotene (peak III) and (*e*) 15-*cis*-β-carotene (peak IV). The sum of all β-carotene peaks is considered as 100%. UV–visible spectra are depicted in the insets. An ANOVA was performed to determine significance (*n* = 3). *p < 0.05, **p < 0.01, ***p < 0.001, ****p < 0.0001. Error bars represent the s.d.

β-Carotene occurs naturally in four stereo-configurations: all-*trans*-, 9-*cis*-, 13-*cis*- and 15-*cis*. We performed *in vitro* assays with the aforementioned β-carotene geometric isomers, using crude lysates of thioredoxin-*Ca*D27-expressing BL21 *E. coli* cells with pGro7 plasmid, which encodes chaperones that improve protein folding, as described by Abuauf *et al*. [13] and Yang *et al.* [17]. The incubation of thioredoxin-*Ca*D27 with all-*trans*-β-carotene changed the content of *cis* isomers (figure 1*a* and electronic supplementary material, table S1), consistent with the *in vivo* assays (electronic supplementary material, figure S1a). The incubation of thioredoxin-*Ca*D27 with 9-*cis*-β-carotene showed results different from those obtained with D27 and D27-Like1 from higher plants [13,17], as *Ca*D27 did not convert 9-*cis*-β-carotene into the all-*trans*-isomer (figure 1*a,b*). Remarkably, thioredoxin-*Ca*D27 converted 13-*cis*-β-carotene into 9-*cis*-β-carotene, increasing the 9-*cis*/13-*cis* ratio from approximately 5% (electronic supplementary material, table S2) to 11% (figure 1*d*) in the control incubation. In addition, we observed the conversion of 15-*cis*-β-carotene into 9-*cis*-β-carotene (figure 1*e*). We also tested the activity of thioredoxin-*Ca*D27 protein preparation on different carotenoids: all-*trans*-lutein, -violaxanthin, -neoxanthin and α-carotene. However, no isomerization activity was observed (electronic supplementary material, figure S3a–d).

### (b) Phylogenetic analysis of D27 orthologues

We surveyed D27 genes from 204 genomes of cyanobacterial species registered in RefSeq (https://www.ncbi.nlm.nih.gov/refseq/) as representative assemblies and found 22 cyanobacteria species containing D27 genes (electronic supplementary material, table S3). To investigate the evolutionary relationships of the D27 genes between cyanobacteria and green photosynthetic eukaryotes in Archaeplastida, we conducted a phylogenetic analysis.

**Figure 2.**
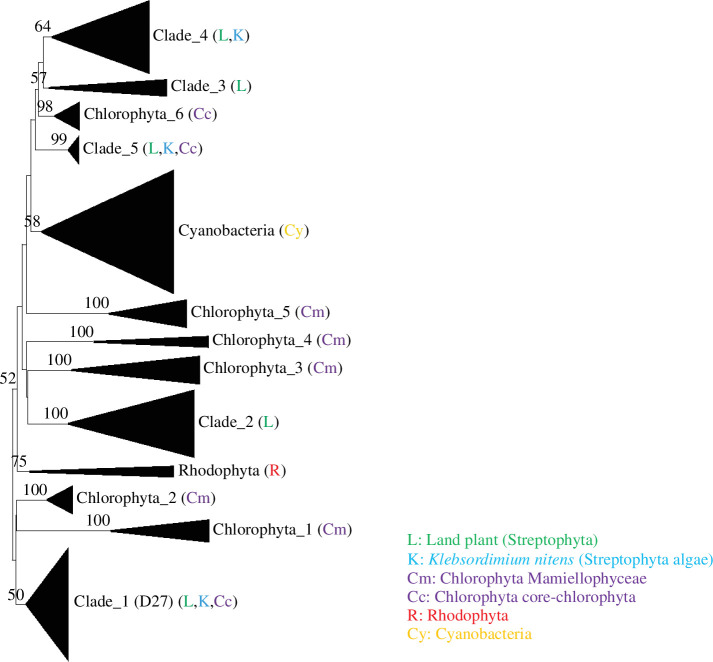
Non-root neighbour-joining tree of D27 orthologues in Archaeplastida. Subtrees designated with black triangles are depicted in electronic supplementary material, figure S4. Information on sequences used for this study is given in electronic supplementary material, table S3. Bootstrap values of more than 50% are shown.

Our phylogenetic tree showed 13 clusters supported with bootstrap values greater than 50% (Clades 1–5, Chlorophyta 1–6, Rhodophyta and Cyanobacteria) (figure 2 and electronic supplementary material, figure S4). Species of land plants and Chlorophyta species contain several orthologues in their genomes (electronic supplementary material, table S3). Land plants generally have five orthologues (Clades 1–5). D27 and D27-Like2 from *Arabidopsis thaliana* (Ar.01 and Ar.04) and *Oryza sativa* D27 isoforms (Os.01 and Os.02) were included in Clade 1, whereas *A. thaliana* D27-Like1 protein (Ar.03) belonged to Clade 4. Orthologues in Clades 1 and 4 were conserved in all land plant species tested in this study. On the other hand, Clade 2 orthologues were found only in seed plants; Clade 3 orthologues were only conserved in moss and fern species, namely *Physcomitrella patens*, *Marchantia polymorpha* and *Selaginella moellendorfii*; and Clade 5 orthologues were conserved in moss species (*M. polymorpha* and *P. patens*). In Chlorophyta species, seven orthologues were identified in the phylogenetic tree. However, the orthologous conserved genomes differed according to the taxonomic groups of cores Chlorophyta and Mamiellophyceae. Core Chlorophyta species possessed Clades 1 and 5 and Chlorophyta 6 orthologues, whereas Chlorophyta 1, 2, 3, 4 and 5 orthologues were conserved in Mamiellophyceae species. Moreover, the D27 genes were conserved as a single copy in the genomes of Rhodophyta and Cyanobacteria species. All sequences of cyanobacteria were clustered into one, and sequences of other taxa, such as those of Rhodophyta, were not included in the phylogenetic tree. In addition, no clear relationships supported by bootstrap values of over 50% were observed among the orthologous groups of cyanobacteria or Rhodophyta and any other orthologous group designated in the phylogenetic tree. However, these two orthologues were included in a large cluster supported by a bootstrap value of 52% with other orthologous groups, except for Clade 1 and Chlorophyta 1 and 2.

## 3. Discussion

Carotenoid metabolism is a fundamental requirement for photosynthesis, light harvesting and photoprotection in photosynthetic organisms [2,3] and may be the original function in the evolution of CCDs and D27 proteins. Carotenoids are excellent sensors of oxidative stress because of their susceptibility to oxidation, and apocarotenoids may have evolved as signals of oxidation [3,4,20]. The formation of apocarotenoids may be regulated by the development of cleavage enzymes, mainly the members of the CCD family, which target specific double bonds in defined carotenoid or apocarotenoid substrates [3]. Research has shown an increase in CCD-catalysed apocarotenoid formation in cyanobacteria under stressful conditions [32,33]. Apocarotenoid stress signals, including β-ionone and β-cyclocitral, have been detected in cyanobacteria and algae [34–36].

Recently, SLs have been characterized as plant hormones with diverse functions within and outside the plant [4,20,24,32,33,37]. SLs are a group of structurally different compounds divided into canonical and non-canonical types [3,4,20,23,24,28]. Canonical SLs are more important for rhizospheric communication and are not the main determinants of shoot branching, a functional characteristic of SLs [16,38]. The origin of SLs is under active debate, that is, whether they appeared first as a true hormone signal early in evolution or as an evolutionary component developed by plants during their transition to land in the form of a rhizosphere signalling molecule [16,19,20,23,26,32,33]. SL analysis has demonstrated the wide occurrence of SL biosynthesis in plants and their presence in angiosperms, gymnosperms, lycophyte mosses, and liverworts [4,8,16,24]. Characterization of the SL core genes (*D27*, *CCD7* and *CCD8*) in different species provided insight into the evolution of SL biosynthesis. However, the recent characterization of D27-like proteins has complicated this equation [17]. Gene duplication is a characteristic of gene evolution and is required for functional diversification [20,32,33]. Moreover, the phylogenetic analysis of *CCD7* sequences from chlorophytes, charophytes and major land plants did not reveal any gene duplications [20,32,33]. Several phylogenetic models have shown an ancestral lineage of core genes, but some genes are missing in certain taxa [23,24,32,33]. Interestingly, *CCD7* and *CCD8* are arranged in a monophyletic clade in land plants, suggesting that these two *CCDs* evolved from a single ancestral orthologue. Orthologous *CCD* genes classified as this ancestor are present in the genomes of algae and other photosynthetic eukaryotes [20,24,32,33]. The distribution of *CCD* genes appears inconsistent, as several model species lack SL synthesis enzymes. For example, *M. polymorpha* lacks *CCD8* and *MAX1*, whereas *P. patens* lacks MAX1 [20,24,32,33]. However, *P. patens* contains *D27*, *D14* and *MAX2* [20,32,33]. An investigation of *P. patens* with disrupted *CCD8* indicated the role of SLs in gametophytic development, demonstrating their versatility and function in development [37].

Nevertheless, it appears that SL biosynthesis and signalling systems are conserved among land plants; however, this is not the case for algae and other photosynthetic eukaryotes [8,20,23,32,33]. The SL signalling pathway is similar to that of karrikins. Karrikins are smoke-derived compounds that trigger seed germination in non-parasitic plants and exert the activity of an unidentified plant signalling molecule [3,33,39]. The karrikin receptor (KAI2) is phylogenetically close to the SL receptor D14 [20,33,39]. Remarkably, D14 is absent in mosses and liverworts, whereas KAI2 is present in all land plants, suggesting that during land plant evolution, D14 emerged as a result of *KAI2* gene duplication [20,23,32,33,39].

Our genome survey showed that *D27* genes are present in the genomes of all eukaryotic species of Archaeplastida. Previous studies have divided *D27* genes into three orthologous groups [8,24,33]. The phylogenetic analysis conducted in this study showed that the *D27* genes of Chlorophyta can be categorized into eight orthologous groups, six of which are unique to this taxonomic group (class level). We suggested that *D27* genes have diverged independently in Chlorophyta. In contrast, three orthologues were identified in the genome of *Klebsormidium nitens*. Each of these orthologues belongs to the orthologous groups of the *D27* in land plants. *K. nitens* is an algal species belonging to Streptophyta and is considered to have ancestral characteristics of land plants belonging to Archaeplastida [40]. Our results suggest that the three genes of *K. nitens* might be ancestral types of the three orthologues (Clades 1, 4 and 5) of *D27* in land plants. In contrast, in Rhodophyta, a taxon that branched earlier than Chlorophyta in evolutionary history, *D27* genes seem to be conserved as a single copy in their genomes, suggesting that Rhodophyta species have only one orthologue. Although the diversification of *D27* orthologues was observed in Chlorophyta and land plants in this study, the *D27* gene is expected to be present in the genome of the ancestor of Archaeplastida eukaryotes.

Cyanobacterial *D27* genes formed an independent cluster in the phylogenetic tree, and bootstrap analysis did not support the phylogenetic relationships between cyanobacterial *D27* genes and those of other taxonomic groups depicted in the phylogenetic tree. The phylogenetic tree also showed that the cluster representing the cyanobacterial *D27* orthologous group formed a large cluster with other groups such as Clades 2 and 3, and Chlorophyta 3. Additionally, Clade 1, which included *A. thaliana* and *O. sativa D27*, was not included in this large cluster, suggesting that *D27 sensu stricto* is a relatively new orthologous group generated through the evolution of land plants. Although some analyses placed cyanobacterial *D27* in the *D27-Like1* clade [24], our study does not support this. Our results also revealed that cyanobacterial *D27* genes were present in their genomes as single copies, indicating that gene duplication events did not occur in cyanobacterial genomes during evolution. This suggests that cyanobacteria retain only one *D27* orthologue, allowing us to predict that functional differentiations are less likely to occur in the evolution of cyanobacterial *D27* genes.

*D27* genes were found in all genomes of the Archaeplastida eukaryotes used in this study, whereas in cyanobacteria, they were found in species of a certain range of taxonomic groups. Several studies have suggested that cyanobacterial taxa possessing *D27* genes tend to branch relatively early in the phylogenetic tree [40], indicating that *D27* genes disappeared at a later stage in the evolution of cyanobacteria. We did not find *D27* genes in prokaryotes other than cyanobacteria using Blast search (https://blast.ncbi.nlm.nih.gov/Blast.cgi) (electronic supplementary material, table S4). Information regarding the cyanobacterial *D27* genes and the species maintaining it would be a powerful hint in elucidating the ancestor of chloroplasts.

*Ca*D27 could be involved in the biosynthesis of an ancestral apocarotenoid signalling molecule that arises from 9-*cis*-configured carotenoids, as in ABA and SLs. In this study, we demonstrated that *Ca*D27 is a β-carotene isomerase with a preference for *cis*/*cis* isomerization activity and formation of 9-*cis*-β-carotene. Previous studies on rice, *Arabidopsis* D27 and *Arabidopsis* D27-Like1 have shown that these enzymes catalyse the reversible isomerization of all-*trans*-β-carotene into 9-*cis*-β-carotene [12,17,25,27]. In contrast, *Ca*D27 does not catalyse this reversible isomerization; we only detected the isomerization of all-*trans*-β-carotene into 9-*cis*, and 13-*cis*-β-carotene, but not of 9-*cis*- into all-*trans*-β-carotene. We also observed the unidirectional isomerization from 13-*cis*- and 15-*cis-* to 9-*cis*-β-carotene, indicating the isomerization activity of *Ca*D27 at C13–C14 and C15–C15′ double bonds in β-carotene (figure 1*a–e*). This *cis*/*cis* β-carotene isomerization activity was consistent with that reported for *At*D27-Like1 [17,27], in the same way as *At*D27-Like1 incapability of isomerizing xanthophylls regardless of their stereo-configuration. (electronic supplementary material, figure S5). Interestingly, the expression of *Ca*D27 in neurosporene-accumulating *E. coli* revealed isomerization activity converting all-*trans*-neurosporene to 9-*cis-* and 15-*cis*-neurosporene, which is similar to saffron D27 [29].

We propose that *Ca*D27 maintains the *cis*-β-carotene pool. However, further analysis is warranted to elucidate the possible compounds that can be synthesized from this pool. We suggest that an apocarotenoid can be the molecule formed from the *cis*-β-carotene products, particularly the 9-*cis*-β-carotene. The 9-*cis*-β-carotene cleavage product, 9-*cis*-β-apo-11-carotenal, was recently shown to act as an ABA precursor in an ABA1-independent biosynthetic pathway [41]. Whether cyanobacteria containing D27 enzymes produce ABA or a different, unidentified regulatory metabolite originating from *cis*-configured β-carotene remains unclear. However, the activity of cyanobacterial D27 enzymes is assumed to be related to maintaining a balance of β-carotene stereoisomers or regulating carotenoid biosynthesis.

## 4. Experimental procedures

### (a) Carotenoid substrates used in the *in vivo*/*in vitro* assays

All carotenoid substrates used were as described by Yang *et al*. [17].

### (b) *In vitro* assays with cyanobacterial D27 enzyme

BL21(DE3) *E. coli* competent cells transformed with pGro7 (Takara Bio, Shiga, Japan), harbouring the chaperones that assist in the folding and assembly of target proteins, were transformed with the empty plasmids pThio-Dan1 [42], pThio-Dan1-*Ca*D27(-cTP), pThio-Dan1-*At*D27(-cTP) and pThio-Dan1-*At*D27-Like1(-cTP) (elecectronic supplementary material, appendix S1). The *in vitro* assays were performed as described by Yang *et al*. [17].

### (c) *In vivo* assays with cyanobacterial D27 enzyme

The empty vectors pThio-Dan1 [42], pThio-Dan1-*Ca*D27(-cTP) were transformed into transgenic *E. coli* strains that accumulate β-carotene, lycopene and zeaxanthin [30,31]. All of the *in vivo* assays were performed as described by Yang *et al*. [17].

### (d) Ultra-high performance liquid chromatography analysis

Ultra-high performance liquid chromatography (UHPLC) analysis and specifications were performed as described by Yang *et al* [17].

### (e) Plasmid construction

The pThio-Dan1-*At*D27 was reported previously [13]. The pThio-Dan1-*Ca*D27 was generated according to [13]. Synthetic *At*D27-Like1 cDNA after removal of cTP was obtained according to [17] (electronic supplementary material, appendix S1).

### (f) Statistical analysis

The analyses were conducted using Prism, version 10.2 (GraphPad Software, San Diego, CA, USA) and Excel 2016 (Microsoft Corporation, Redmond, WA, USA). For all experiments, at least three replicates were measured in parallel and data are presented as the mean ± s.d. Three independent experiments confirmed all presented data. Statistical significance was determined by an ANOVA using multiplicity-adjusted *p* values.

### (g) Phylogenetic analysis

We obtained 204 genome assemblies (a protein FASTA format) of cyanobacterial species registered in RefSeq as representative assemblies. We also selected 21 species genome assemblies (a protein FASTA format) of Archaeplastida species in the RefSeq assembly database belonging to Rhodophyta (2 species), Chlorophyta (11 species), Streptophyta algae (*K. nitens*) and land plants (moss, 2 species; fern, 1 species, Spermatophyta, 4 species) to find D27 orthologues (electronic supplementary material, table S3).

Since no genome assemblies were available for *Crocus sativus*, we used all the protein sequences of this species registered in the NCBI database for the D27 orthologue survey. All protein sequences obtained from these 225 species were functionally annotated with the Pfam-A database [43], and the sequences possessing the functional domain designated as PF13225 were supported with *e*-values smaller than 1.0*e*−10. After removing the duplicated protein sequences, we obtained 94 D27 orthologue sequences.

The sequence alignment was built with MAFFT v. 7.487 software (https://mafft.cbrc.jp/alignment/software/) using the full length of these sequences, and the neighbour-joining tree was constructed with MEGA 10 software (https://www.megasoftware.net/). The bootstrap precedure was replicated 1000 times.

## Data Availability

All the information is included in the online electronic supplementary material that accompanies this article [44].
